# The Relationship of Docosahexaenoic Acid (DHA) with Learning and Behavior in Healthy Children: A Review

**DOI:** 10.3390/nu5072777

**Published:** 2013-07-19

**Authors:** Connye N. Kuratko, Erin Cernkovich Barrett, Edward B. Nelson, Salem Norman

**Affiliations:** DSM Nutritional Products, 6480 Dobbin Road Columbia, MD 21045, USA; E-Mails: erin.barrett@dsm.com (E.C.B.); ed.nelson@dsm.com (E.B.N.); norman.salem@dsm.com (N.S.)

**Keywords:** docosahexaenoic acid, children, learning, behavior, school performance

## Abstract

Childhood is a period of brain growth and maturation. The long chain omega-3 fatty acid, docosahexaenoic acid (DHA), is a major lipid in the brain recognized as essential for normal brain function. In animals, low brain DHA results in impaired learning and behavior. In infants, DHA is important for optimal visual and cognitive development. The usual intake of DHA among toddlers and children is low and some studies show improvements in cognition and behavior as the result of supplementation with polyunsaturated fatty acids including DHA. The purpose of this review was to identify and evaluate current knowledge regarding the relationship of DHA with measures of learning and behavior in healthy school-age children. A systematic search of the literature identified 15 relevant publications for review. The search found studies which were diverse in purpose and design and without consistent conclusions regarding the treatment effect of DHA intake or biomarker status on specific cognitive tests. However, studies of brain activity reported benefits of DHA supplementation and over half of the studies reported a favorable role for DHA or long chain omega-3 fatty acids in at least one area of cognition or behavior. Studies also suggested an important role for DHA in school performance.

## 1. Introduction

The period from birth to 2 years of age is considered the primary growth phase for the human brain when measured in terms of brain weight. However, certain areas of the brain are not fully developed by the age of two, and development as well as growth continues throughout childhood and adolescence [1]. Myelination of brain frontal lobes begins as early as 6 months of age and continues throughout childhood and adolescence with spurts of development identified at 2 years of age, 7–9 years of age, and during mid-adolescence [1]. Tissue content of the long chain, omega-3 fatty acid (*n*-3 LC-PUFA) docosahexaenoic acid (DHA, 22:6*n*-3) is important for this development. DHA-rich frontal lobes are thought to be responsible for executive and higher-order cognitive activities such as planning, problem solving, and focused attention [2]. Investigators report an association of these prefrontal cortex structures with the limbic system, where the development of high-level cognitive function also correspond to a child’s social, emotional and behavioral development [3,4].

Many components of the diet are known to affect cognition and influence learning. DHA in particular is recognized as essential for normal brain function. DHA is the principal omega-3 fatty acid in brain gray matter representing about 15% of all fatty acids in the human frontal cortex [5] and is known to affect neurotransmitter pathways, synaptic transmission, and signal transduction. Its multiple double bonds and unique structure allow DHA to impart a disorder to membranes which allows for effective cell signaling [6,7]. Studies in animals and humans show that adequate levels of DHA in neural membranes are important for cortical astrocyte maturation and vascular coupling, and for cortical glucose uptake and metabolism [8,9,10,11,12,13]. In addition, certain metabolites of DHA are bioactive molecules which protect tissues from oxidative injury and stress [14,15,16,17]. In animals, low brain DHA results in changes in behavior and is associated with learning problems and memory deficits [18]. In humans, studies at various life stages indicate that DHA supports normal IQ [19] and preserves visuo-spatial learning and memory [20]. For the brain and retina, therefore, it is clear that adequate DHA composition allows for optimal function [12]. Low blood levels of *n*-3 LC-PUFA, as well as high *n*-6 to *n*-3 ratios are reported in children with certain developmental and behavioral disorders such as attention deficit hyperactivity disorder (ADHD), dyslexia, or dyspraxia. Although inconsistent, various fatty acid supplementation strategies, some of which included DHA, proved to benefit measures of learning and behavior in these children. Some investigators even report clinical signs of fatty acid deficiency in these children which respond to *n*-3 LC-PUFA supplementation [21,22]. Reasons for this low omega-3 status are not fully understood, but could include disorders of fatty acid metabolizing enzymes and pathways which are unique to the disease. However, the explanation could also include a childhood history of inadequate *n*-3 fatty acid intake [23,24,25]. This brings to question whether poor *n*-3 LC-PUFA status in otherwise healthy children might also impact learning, memory, and behavior. If so, could improvement in *n*-3 LC-PUFA status, and DHA status in particular, improve learning and performance in healthy children? The current review was conducted to assess current knowledge regarding the relationship of DHA with learning (including such measures as reading and spelling), memory, and behavior in healthy children.

## 2. Experimental Section

All observational studies evaluating DHA intake or status as well as randomized controlled trials (RCT) evaluating DHA supplementation (with or without the addition of other fatty acids and nutrients) in healthy children, age 4 to 18 years, were considered for review. Eligible outcomes included all measures of cognitive function, learning, and behavior, with special interest in school performance or assessments such as those for reading, spelling, or listening comprehension. For inclusion, the publication had to quantify DHA in the diet or supplement, or include DHA as a biomarker measured in tissue or blood. Analyses had to report neurocognitive outcomes in relation to a concurrent indicator of DHA intake or status. Studies were included if subjects were generally described as healthy, without diagnosis of a neuropsychological condition, and participating in the available mainstream school setting. Studies in children with known deficiencies in nutrients other than DHA were included if no psychological or learning disabilities were apparent. Studies in children diagnosed with a learning disability or with special educational needs were excluded. Many of the authors of the studies included in this review performed multiple analyses due to an interest in several outcomes and various subgroups. While it is true that the more analyses that are done, the more likely that some of them will be statistically significant by chance, the correction for multiple comparisons applied by the study authors was not consistent. In order to provide a comprehensive review of the literature and avoid re-interpretation of the reported outcomes, no studies were excluded due to method of statistical assessment.

Studies which included α-linolenic acid (ALA, 18:3 *n*-3) or sources of ALA as the only treatment intervention were excluded. However, studies which used micronutrient or fatty acid mixtures were included if the formulation included DHA. Whenever multiple publications reported unique analyses pertaining to the same subjects, all publications were retained. The database of PubMed was searched in November, 2012 using a predefined algorithm. References from pertinent systematic reviews were screened for potential articles that might have been missed in the search.

Search results were sorted by an investigator at the title/abstract level, eliminating inappropriate articles. Two investigators independently reviewed the full papers of the remaining studies to determine the final accepted list. The search criteria were built on the following PubMed Mesh terms:

((“Fatty Acids, Omega-3” [Mesh] OR “Fish Oils” [Mesh]) OR “Docosahexaenoic Acids” [Mesh]) OR “Eicosapentaenoic Acid” [Mesh]

And

(((((“Learning” [Mesh] OR “Memory” [Mesh]) OR “Reading” [Mesh]) OR “Students” [Mesh]) OR “Schools” [Mesh]) OR “Education” [Mesh]) OR “Educational Status” [Mesh]

And

Filters activated: English, Preschool Child: 2–5 years, Child: 6–12 years, Adolescent: 13–18 years.

Search terms to select the populations of interest included: All infant birth-23 months; Preschool Child 2–5 years, Child 6–12 years.

Exclusion criteria included subject populations of children diagnosed with or a history of ADHD, dyslexia, autism, dyspraxia, or learning disabilities requiring special education, or medication use for any of these conditions. Other exclusions included fish-only interventions without calculation or confirmation of the fatty acids provided; analyses reporting only fish-related outcomes, with no report of fatty acid contribution; reviews or mechanistic papers; outcomes from *in vitro* or animal models; and follow-up studies of prenatal or infant interventions failing to report intake or biomarker data corresponding with outcome assessments in the older child.

## 3. Results

### 3.1. Search Results

Using the above criteria, the PubMed search resulted in 38 citations. Level 1 Screening, based on the abstract excluded 24 articles. Full papers were retrieved for the 14 remaining studies, two of which were excluded. Three additional studies were located from reviewing the references in the retrieved papers. Fifteen articles conducted in healthy children ages 4–14 years met the full criteria for inclusion in this review. Table 1, Table 2 include a list of the final accepted studies with reported outcomes. Table 3 is a summary of DHA biomarker data reported by study.

Three studies were observational in design [26,27,28]. Bakker *et al.* [26] and Boucher *et al*. [27] provided a prospective analysis of cohorts identified in infancy and followed through age 7 and through 10–13 years, respectively. The cross-sectional study by Kirby *et al*. [28] reported correlations between cheek cell fatty acids, including DHA, with relevant cognitive and behavioral outcomes.

The remaining 12 studies were randomized, controlled trials of varied purpose and design. The sources of DHA administered as the intervention included triglycerides from fish oil and algal oil. The oils were given as supplements in capsules or chewable form, or were incorporated into fortified foods. Experimental designs often included additional nutrients, such as vitamins, minerals, or other fatty acids. In most of the studies, the placebo consisted of corn, soy, or olive oil, or a combination of those vegetable oils. Daily doses of DHA ranged from 88 to 1200 mg per day.

**Table 1:** Observational studies reporting docosahexaenoic acid (DHA)-related outcomes of cognition and behavior in children.

Reference	Study Design	Participants	Outcome Measures	Results	
				Cognition	Behavior
Boucher *et al*., 2011 [27]	Prospective	*n* = 154 School-aged children (10–13 years) with available cord blood samples	**Cognition** Digit span forward subtest of the Wechsler Intelligence Scales for Children (WISC-IV) The California Verbal Learning Test Children’s Version (CVLT) Continuous Recognition Task (CRT) Event-related potentials (ERPs) were acquired via electroencephalogram recordings while the CRT was performed **Biological Samples** Fatty acid composition of umbilical cord blood Fatty acid composition of child blood	Neuropsychological Assessments Cord blood DHA levels were positively associated with performance on the digit span forward test Cord blood DHA levels were positively associated with performance on the CVLT Electroencephalogram Recordings Higher cord DHA levels were associated with a shorter latency of the FN400 component and a larger amplitude of the LPC Higher current DHA levels were associated with a greater FN400 amplitude	
Kirby *et al*., 2010 [28]	Cross-Sectional	*n* = 411 School-aged children (8–10 years)	**Cognition** Kaufman Brief Intelligence Test (KBIT-2) Working Memory Test Battery for Children (WMTB-C) Wechsler Individual Achievement Test (WIAT-II) Test of Everyday Attention for Children (TEA-Ch) Matching Familiar Figures Task (MFFT) **Behavior** Swanson, Nolan, and Pelham (SNAP-IV; parents/guardians and teachers) Strengths and Difficulties Questionnaire (SDQ; parents/guardians and teachers) Developmental Coordination Disorder Questionnaire (DCDQ; parents/guardians) **Biological Samples** Cheek cell samples for measurement of fatty acid composition	Cheek cell DHA levels were positively associated with non-verbal IQ (KBIT-2)	Cheek cell DHA levels were negatively associated with teacher rated hyperactivity and total difficulties (SDQ)
Bakker *et al*., 2003 [26]	Prospective	*n* = 306 School-aged children (7 years of age) with available cord blood samples	**Cognition** Kaufman Assessment Battery for Children (K-ABC) **Biological Samples** Fatty acid composition of umbilical cord blood Fatty acid composition of child blood at age 7	There was no association between cord blood DHA levels and cognitive performance (K-ABC) There was no association between plasma DHA at age 7 and cognitive performance (K-ABC)	

Abbreviations: California Verbal Learning Test (CVLT), Continuous Recognition Task (CRT), Developmental Coordination Disorder Questionnaire (DCDQ), Event-Related Potentials (ERP), Kaufman Assessment Battery for Children (K-ABC), Kaufman Brief Intelligence Test (KBIT), Matching Familiar Figures Task (MFFT), Test of Everyday Attention for Children (TEA-Ch), Strengths Difficulties Questionnaire (SDQ), Swanson, Nolan, and Pelham (SNAP), Wechsler Individual Achievement Test (WIAT), Wechsler Intelligence Scales for Children (WISC), Working Memory Test Battery for Children (WMTB-C).

**Table 2:** Clinical trials of DHA supplementation on outcomes of cognition and behavior in children.

Reference	Study Design	Participants	Intervention	Time (week)	Outcome Measures	Results	
						Cognition	Behavior
Baumgartner *et al*., 2012 [29]	RCT	**Randomized,** *n* = 321 School-aged children with iron deficiency (6–11 years) Completed cognitive follow up, *n* = 288 Placebo, *n* = 73 Iron, *n* = 70 DHA/EPA, *n* = 72 Iron + DHA/EPA, *n* = 73	1. Placebo 2. Iron: 50 mg/day 3. DHA/EPA: 420 mg/80 mg/day 4. Iron + DHA/EPA: 420 mg/80 mg/day NOTE: Supplements were provided 4 days/week on school days only. Overall 4.8 g iron (45.7 mg/day), 41.2 g DHA (392 mg/day), and 7.8 g EPA (74 mg/day) were provided	38	**Cognition** Kaufman Assessment Battery (K-ABC-II) Hopkins Verbal Learning Test (HVLT)	**K-ABC-II** In all groups, tests of learning abilities (Atlantis, Atlantis Delayed) and test of simultaneous processing (Triangles) improved significantly (time-effect) Scores for sequential processing (Hand movement) decreased in all but the placebo plus DHA/EPA group Among all children, there were no significant intervention effectsThere was a significant effect of DHA/EPA for lower Atlantis test scores in children with anemia There was a significant negative effect of DHA/EPA supplementation on Atlantis Delayed test performance in girls. **HVLT** Among all children, DHA/EPA supplementation had no effect on HVLT scores Girls supplemented with DHA/EPA alone recalled significantly more words than girls given the combined treatments or placebo	
Dalton *et al*., 2009 [30]	RCT	**Randomized,** *n* = 183 Low-income, marginally nourished school aged-children (7–9 years) **Per-Protocol,** *n* = 155 Control bread spread, *n* = 78 Fish-flour bread spread, *n* = 77	1. Control spread 2. Fish flour spread: ~892 mg DHA/week NOTE: The spread was provided on school days only and not on weekends or during school holidays	24	**Cognition** Hopkins Verbal Learning Test (HVLT) Reading Spelling	**Verbal Learning** Fish flour spread improved verbal learning ability including identification of true positive (*p* = 0.0191), and false negatives (*p* = 0.0075) **Spelling** Fish flour spread protected against declines in spelling	
Hamazaki *et al*., 2008 [31]	RCT	**Randomized,** *n* = 233 Healthy, school-aged children (9–14 years) **Per-Protocol, ***n* = 189 Placebo tablets, *n* = 96 Fish oil, *n* = 93	1. Placebo capsules 2. Fish oil: 650 mg DHA/day + 100 mg EPA/day	12	**Behavior** Hostility-Aggression Questionnaire for Children (HAQ-C) Barratt Impulsiveness Scale (BIS-11) School attendance		**Aggression/Impulsiveness** Fish oil had no effect on aggression or impulsiveness **School Attendance** Fish oil improved school attendance rate (*p* = 0.003)
Itomura *et al*., 2005 [32]	RCT	**Randomized, *n* = 179** Healthy, school-aged children (9–12 years) **Per-Protocol, *n* = 166** Control foods, *n* = 83 Fish oil fortified foods, *n* = 83	1. Control foods 2. Fortified foods: ~514 mg DHA/day ~120 mg EPA/day	12	**Behavior** Hostility-Aggression Questionnaire for Children (HAQ-C) Picture Frustration (PF) Study Diagnostic questionnaires for ADHD		**Aggression** Among females, fish oil protected against increases in aggression as assessed by the HAQ-C (*p* = 0.008) Among males, aggression against others as assessed by the PF study, increased in the fish oil group but not in the control group **Impulsivity (DSM-IV)** Among females, fish-oil reduced impulsivity (*p* = 0.008)
Kennedy *et al*., 2009 [33]	RCT	**Randomized** *n* = 90 Healthy, school-aged children (10–12 years) **Per-Protocol** *n* = 88 Placebo tablets, *n* = 30 Low-dose algal DHA, *n* = 28 High-dose algal DHA, *n* = 30	Placebo capsules Algal DHA: 400 mg/day Algal DHA: 1000 mg/day	8	**Cognition** Cognitive Drug Research (CDR) battery Internet Battery	**CDR** Except for the word recognition task, DHA had no effect on cognition For the word recognition task, children given low-dose DHA performed significantly faster (*p* < 0.05) while children given high-dose DHA performed significantly slower (*p* < 0.05)	**Internet battery** Except for the visual analog ratings of “relaxed”, DHA had no effect on cognition For ratings of “relaxed”, children given low and high dose DHA rated themselves as “more relaxed”
Kirby *et al*., 2010 [34]	RCT	**Randomized,** *n* = 450 Healthy, school-aged children (8–10 years) **Per-Protocol,** *n* = 348 Placebo tablets, *n* = 177 Fish oil + micronutrients, *n* = 171 NOTE: After 16 weeks of supplementation, all children received the active supplement for an additional 8 weeks (one-way cross-over)	Placebo capsules Fish oil: 400 mg DHA/day 56 mg EPA/day 800 vitamin A 60 g vitamin C 5.0 μg vitamin D 3.0 mg vitamin E	16	**Cognition** Kaufman Brief Intelligence Test (KBIT-II) Wechsler Individual Achievement Test (WIAT-II) Working Memory Test Battery for Children (WMTB-C) Test of Everyday Attention for Children (TEA-Ch) Matching Familiar Figures Task (MFFT) Computerized Penmanship Evaluation Tool (ComPET) **Behavior** Swanson, Nolan, and Pelham (SNAP-IV) Strengths and Difficulties Questionnaires (SDQ)	In the ITT analysis, fish oil had no effect on IQ, reading and spelling, working memory, attention, visual attention, impulsivity, or handwriting process Among compliant children (≥80% of dosing requirement), fish oil improved visual attention and impulsivity as assessed by the MFFT (*p* < 0.001)	**SDQ** Among all children, teacher rated difficulties score improved in the control group but not in the fish oil group (*p* < 0.001) Among compliant children (≥80% of dosing requirement), fish oil protected against declines in parent rated prosocial behavior (*p* = 0.001)
McNamara *et al*., 2010 [12]	RCT	**Randomized** *n* = 38 Healthy, school-aged boys (8–10 years) **Per-Protocol** *n* = 33 Placebo tablets, *n* = 10 Low-dose algal DHA, *n* = 10 High-dose algal DHA, *n* = 13	Placebo capsules Algal DHA: 400 mg/day Algal DHA: 1200 mg/day	8	**Cognition** Functional magnetic resonance imaging (fMRI) Identical-Pairs version of the Continuous Performance Task (CPT-IP)	**Whole-brain activation patterns during performance of sustained attention tasks** Low-dose DHA improved brain activation (increased activation in the dorsolateral prefrontal cortex, decreased activation in the occipital cortex) High-dose DHA improved brain activation (increased activation in the dorsolateral prefrontal cortex, decreased activation in the cerebellar cortex) Erythrocyte DHA positively correlated with dorsolateral prefrontal cortex activation **Sustained attention** Low- and high-dose DHA had no effect on sustained attention performance Erythrocyte DHA inversely correlated with reaction time on the sustained attention test (*p* = 0.02)	
Muthayya *et al*., 2009 [35]	RCT	**Randomized,** *n* = 598 Low income, marginally nourished school-aged children (6–10 years) **Per-Protocol,** *n* = 550 Low micronutrient/low omega-3, *n* = 140 Low micronutrient/high omega-3, *n* = 139 High micronutrient/low omega-3, *n* = 133 High micronutrient/high omega-3, *n* = 138	**Low micronutrient** Vitamin A: 75 μg RE/day Riboflavin: 0.14 mg/day Vitamin B6: 0.15 mg/day Vitamin B12: 0.27 μg/day Folate: 45 μg/day Vitamin C: 5.25 mg/day Calcium: 105 mg/day Iodine: 15 μg/day Iron: 2.7 mg/day Zinc: 1.7 mg/day **High micronutrient** Vitamin A: 500 μg RE/day Riboflavin: 0.9 mg/day Vitamin B6: 1 mg/day Vitamin B12: 1.8 μg/day Folate: 300 μg/day Vitamin C: 227.1 mg/day Calcium: 231 mg/day Iodine: 100 μg/day Iron: 18 mg/day Zinc: 10.5 mg/day **Low omega-3** Total omega-3: 0.14 g/day ALA: 0.14 g/day DHA: 0 g/day **High omega-3** Total omega-3: 1.03 g/day ALA: 0.93 g/day DHA: 0.10 g/ day	52	**Cognition** Kaufman Assessment Battery for Children (K-ABC) Wechsler Intelligence Scales for Children (WISC-R and WISC-4) Rey Auditory Verbal Learning Test (RAVLT) Neuropsychological Assessment tool (NEPSY) Number cancellation	All four groups improved in short-term memory, retrieval ability, fluid reasoning, cognitive speediness, and the Mental Processing Index compared with baseline There was no significant differences between the high and the low omega-3 groups on any cognitive measure	
Richardson *et al*., 2012 [36]	RCT	**Randomized** *n* = 362 Healthy, school-aged children (7–9 years), who were underperforming in reading (<33rd% tile) **Per-Protocol** *n* = 359 Placebo tablets, *n* = 180 Algal DHA, *n* = 179	Placebo capsules Algal DHA: 600 mg/day	16	**Cognition** British Ability Scale (BAS-II) **Behavior** Conners’ Parent Rating Scales (CPRS) Conners’ Teacher Rating Scales (CTRS)	**Reading** For children with baseline reading scores ≤33rd% tile, DHA had no effect on reading For children with baseline reading scores ≤20th (*p* = 0.041) and ≤10th (*p* = 0.011) % tiles, DHA improved reading For children with baseline reading scores ≤10th% tile, DHA led to a 1.9 month gain in reading age (*p* = 0.032) **Working memory** DHA had no effect on working memory	For children with baseline reading scores ≤33rd% tile, DHA improved parent-rated behavior including oppositional (*p* = 0.004), hyperactivity (*p* = 0.007), ADHD index (0.042), Global Restless-Impulsive (*p* = 0.001), Global Emotional Liability (*p* = 0.001), Global Index Total (*p* = 0.001), DSM-IV Hyperactive Impulsive (*p* = 0.021), and DSM-IV Total ADHD (*p* = 0.031) scores For children with baseline reading scores ≤33rd% tile, DHA had no effect on teacher rated behavior
Ryan *et al*., 2008 [37]	RCT	**Randomized** *n* = 175 Healthy 4 year olds **Per-Protocol** *n* = 175 Placebo tablets, *n* = 90 Algal DHA, *n* = 85	Placebo capsules Algal DHA: 400 mg/day	16	**Cognition** Leiter-R test of Sustained Attention Peabody Picture Vocabulary Test Day-Night Stroop Test Conners’ Kiddie Continuous Performance Test	In the ITT analysis, DHA had no effect on cognition Among children who provided a blood sample, DHA in whole blood was positively associated with scores on the Peabody Picture Vocabulary Test (*p* = 0.018)	
Sinn *et al*. 2011 [38]	Open label	*n* = 47 School-aged children (3–14 years) in a remote, primarily Indigenous, Northern Territory school **Per-Protocol** *n* = 37 Fish-oil	Fish oil: 558 mg EPA 174 mg DHA 60 mg GLA 10.8 mg vitamin E NOTE: The capsules were consumed on school days with a 1 week break after 5 weeks	12	**Cognition** Wide Range Achievement Test (WRAT) Raven’s Colored Matrices **Behavior** Conners’ Behavior Ratings Scales (CBRS)	Fish oil improved reading (*p* = 0.01) and spelling (*p* = 0.01) as assessed by the WRAT Fish oil improved non-verbal intelligence as assessed by the Raven’s Colored Matrices (*p* < 0.01)	Behavior was not assessed at week 12 (teachers who completed the CBRS at baseline left the school)
The NEMO Study Group 2007 [39]	RCT	**Randomized,** *n* = 396 Well-nourished, school-aged children (6–10 years) from South Australia **Per-Protocol, *n* = 276** Placebo drink, *n* = 71 Flavored drink + micronutrients, *n* = 67 Flavored drink + omega-3s, *n* = 67 Flavored drink + micronutrients and omega-3s, *n* = 71 **Randomized,** *n* = 384 Marginally nourished, school-aged children (6–10 years) from Indonesia **Per-Protocol,** *n* = 367 Placebo drink, n = 88 Flavored drink + micronutrients, *n* = 91 Flavored drink + omega-3s, *n* = 94 Flavored drink + micronutrients and omega-3s, *n* = 94	**Micronutrient mx** Iron: 10 mg Zinc: 5 mg Vitamin A: 400 μg Folate: 150 μg Vitamin B6: 1 mg Vitamin B12: 1.5 μg Vitamin C: 45 mg **Omega-3 mix** DHA: 8 mg EPA: 22 mg NOTE: The fruit flavored drink was consumed 6 days/week	52	**Cognition** *Both Countries* Wechsler Intelligence Scales for Children (WISC-III; digits backwards, coding, block design, vocabulary) Neuropsychological Assessment tool (NEPSY; visual attention, fluency structured and random) Rey Auditory Verbal Learning Test (RAVLT) Wechsler Individual Achievement Test (WIAT-II; mathematical reasoning) *Australia* WIAT-II (reading, spelling) *Indonesia* Neale Analysis of Reading ability		**Australia** Among well-nourished school-aged children, omega-3 fatty acids had no effect on any measure of cognitive function or school performance **Indonesia** Among marginally nourished school-aged children, omega-3 fatty acids had no effect on any measure of cognitive function or school performance

Abbreviations: Barratt Impulsiveness Scale (BIS-II), British Ability Scale (BAS), Cognition Drug Research (CDR), Computerized Penmanship Evaluation Tool (ComPET), Conners’ Behavior Ratings Scales (CBRS), Conners’ Parent Rating Scales (CPRS), Conners’ Teacher Rating Scales (CTRS), Functional Magnetic Resonance Imaging (fMRI), Hostility-Aggression Questionnaire for Children (HAQ-C), Hopkins Verbal Learning Test (HVLT), Identical-Pairs verson of the Continuous Performance Task (CPT-IP), Intention-to-treat (ITT), Kaufman Assessment Battery (K-ABC), Kaufman Brief Intelligence Test (KBIT), Matching Familiar Figures Task (MFFT), Neuropsychological Assessment tool (NEPSY), Picture Frustration (PF), Ray Auditory Verbal Learning Test (RAVLT), Strengths and Difficulties Questionnaire (SDQ), Swanson, Nolan, and Pelham (SNAP), Test of Everyday Attention for Children (TEA-Ch), Wechsler Individual Achievement Test (WIAT), Wechsler Intelligence Scales for Children (WISC), Wide Range Achievement Test (WRAT), Working Memory Test Battery for Children (WMTB-C).

**Table 3:** DHA status reported in clinical trials of DHA supplementation in children.

Reference	Time Point		Placebo	Treatment		Biomarker
Dalton *et al*., 2009 [30]	Baseline					% of total fatty acids (PC in RBCs)
	Endpoint		2.81 ± 0.70	3.71 ± 0.66		
	Baseline					% of total fatty acids (PE in RBCs)
	Endpoint		7.77 ± 2.16	8.98 ± 2.61		
	Baseline					% of total fatty acids (PC + PE in RBCs)
	Endpoint		10.58	12.69		
Hamazaki *et al*., 2008 [31]	Baseline		4.4 ± 0.9	4.4 ± 1.1		% of total fatty acids (phospholipid fraction in RBCs)
	Endpoint		4.9 ± 1.2	7.8 ± 1.1		
Itomura *et al*., 2005 [32]	Baseline		6.4 ± 0.9	6.1 ± 0.9		% of total fatty acids (phospholipid fraction in RBCs)
	Endpoint		6.6 ± 0.9	7.1 ± 1.8		
Kennedy *et al*., 2009 [33]	**NR**					
Kirby *et al*., 2010 [34]	Baseline		0.07 ± 0.007	0.08 ± 0.009		% of total fatty acid methyl esters (buccal cell sample)
	Endpoint		0.20 ± 0.16	0.37 ± 0.23		
Richardson *et al*., 2012 [36]	**NR**					
Ryan *et al*., 2008 [37]	Baseline		1.0 ± 0.34	1.0 ± 0.34		% of total fatty acids (capillary whole blood)
	Endpoint		1.1 ± 0.40	3.3 ± 1.54		
Sinn *et al*., 2011 [38]	**NR**					
Baumgartner *et al*., 2012 [29]		**Placebo**	**Iron**	**DHA/EPA**	**Iron + DHA/EPA**	
	Baseline	3.07 ± 0.69	3.07 ± 0.64	3.05 ± 0.63	3.01 ± 0.58	% of total fatty acids (phospholipid fraction in RBCs)
	Endpoint	3.79 ± 0.96	3.83 ± 1.06	5.94 ± 1.71	5.90 ± 2.11	
McNamara *et al*., 2010 [12]		**Placebo**		**Low Dose Treatment**	**High Dose Treatment**	
	Baseline	3.3 ± 1.3		3.3 ± 1.3	3.3 ± 1.3	% of total fatty acids (RBC)
	Endpoint	2.5 ± 1.6		7.5 ± 1.3	10.3 ± 1.5	
Muthayya *et al*., 2009 [35]		Low Micro/Low Omega-3	Low Micro/High Omega-3	High Micro/Low Omega-3	High Micro/High Omega-3	
	Baseline	3.3 ± 0.9	3.2 ± 0.9	3.2 ± 0.8	3.2 ± 0.7	% of total fatty acids (phospholipid fraction in RBCs)
	Endpoint	3.6 ± 1.0	5.2 ± 1.2	3.6 ± 0.8	5.2 ± 1.2	
The NEMO Study Group2007 [39]		**Placebo**	**Micronutrients**	**DHA/EPA **	**Micronutrients + DHA/EPA**	
	Baseline	33.2 ± 11.0	35.2 ± 10.4	31.1 ± 10.6	32.7 ± 10.3	Plasma (μg/mL)
	Endpoint	38.4 ± 14.0	36.8 ± 8.5	47.4 ± 12.7	47.4 ± 10.7	

Abbreviations: Red blood cells (RBCs), Phosphatidylcholine (PC), Phosphatidylethanolamine (PE), Not reported (NR).

Five of the included studies, or study arms, were conducted in populations of children with multiple nutrient deficiencies [29,30,35,38,39]. It is commonly known that many nutrients other than DHA affect neurological development and cognition in children. Although the question has been raised, it is unknown to what extent cognitive function can be affected based on replenishment of *n*-3 LC-PUFA alone in the case of generalized malnutrition [40]. Further investigation of this question should be considered in future studies. However, since the studies met the pre-defined inclusion criteria for this review by enrolling children without a neuropsychological diagnosis, and analyzing data in a *n*-3 LC-PUFA specific manner, those studies conducted in populations with an increased incidence of malnutrition were included in this review.

The number of subjects varied greatly among the studies, ranging from 33 in the study by McNamara *et al*. [12] to 598 in the study by Muthayya *et al*. [35]. Study duration was also variable and ranged from 2 [33] to 12 months [35,39]. Many of the studies reported results as analyzed both in the “intention to treat” as well as the “per protocol” subject groups, and most reported results as a degree of change from baseline. Compliance was monitored and assessed in all studies and many authors acknowledged the difficulty associated with supplement protocols that often involved subjects, parents, caregivers, and teachers.

The accepted studies included a wide number of assessments of cognition and behavior. All measures were included in this review in order to represent the broadest possible assessment from the limited number of studies. Outcome measures included objective and subjective methodologies including: electrical potential readings and functional magnetic resonance imaging; standardized tests of cognition, including reading, spelling, listening comprehension, memory, attention, and IQ; as well as self- ,parent-, and teacher-assessed behavioral questionnaires. While outcomes were often reported in relation to total omega-3 fatty acids, ALA, or ratios of *n*-3 and *n*-6 fatty acids, the focus of this review was to determine the effect of DHA, specifically, on cognitive and behavioral outcomes.

Perhaps most interesting were the results of DHA supplementation on school performance as reported in seven of the studies [12,27,30,34,36,37,39]. Of the seven studies, five reported that DHA status or supplementation improved measures of school performance including learning ability, reading, and spelling as assessed by sub-tests of cognitive batteries [12,27,30,36,37] while two, in contrast, found no effect [34,39]. Two studies directly assessed brain activity and both reported benefit as the result of DHA intake or status [12,27]. Behavioral outcomes were measured in six studies [28,31,32,33,34,36], with five reporting at least one significant improvement related to DHA supplementation or status [28,32,33,34,36].

### 3.2. Review of Studies

Below are brief summaries of the included studies. Three of the studies were observational in design, seven were clinical studies that provided a lipid intervention, and five were clinical studies that provided other nutrients in addition to a lipid intervention.

#### 3.2.1. Observational Studies

In 2003, Bakker *et al.* [26] reported results of a prospective, longitudinal study investigating the relationship of cognitive performance at 7 years of age with current fatty acid status and fatty acid status at birth. A total of 306 children with available cord blood samples were followed up at age 7 for assessments of cognitive function and achievement using the Kaufman Assessment Battery for Children (K-ABC). The investigators found no association between cord or plasma DHA and cognitive performance in the 7 year olds.

In 2011, Boucher *et al.* [27] reported results from a prospective, longitudinal study examining the effect of prenatal and childhood intake of DHA on memory function in school-aged children from Arctic Quebec. A total of 154 children aged 10–13 years, who had available umbilical cord blood samples, constituted the study population. Visual recognition memory was assessed by event-related potentials (ERPs) recordings during performance of a continuous recognition memory task. Memory was also assessed via a battery of neuropsychological tests including the “Digit span forward” subtest of the Wechsler Intelligence Scales for Children, 4th edition (WISC-IV) and The California Verbal Learning Test—Children’s Version (CVLT). Current blood samples, along with cord blood samples, were analyzed for DHA. The pattern of ERP recordings showed that higher prenatal DHA status positively affected visual information processing and brain activity in later childhood; and, that current DHA intake was positively associated with brain activity during performance of a specific task requiring familiarity processing. Higher cord blood DHA was associated with better performance on both the ‘Digit span forward’ test and the CVLT suggesting better memory development. Neither cord-nor current-DHA blood levels were associated with any of the tasks of continuous recognition memory.

Kirby *et al.* [28] reported results of a cross-sectional study examining the relationship between cheek cell fatty acids and multiple assessments of learning, and behavior in 411 children aged 8–10 years. Assessments included: the Kaufman Brief Intelligence Test (KBIT-2) to assess verbal and non-verbal IQ; the Working Memory Test Battery for Children (WMTB-C) to assess working memory; and, the Wechsler Individual Achievement Test (WIAT-II) to assess reading and spelling ability. Test of Everyday Attention for Children (TEA-Ch) assessed attention capabilities; and, visual attention and impulsivity were assessed using the Matching Familiar Figures Task (MFFT). Aspects of behavior were assessed by parents and teachers using the Swanson, Nolan, and Pelham (SNAP-IV) and the Strengths and Difficulties Questionnaire (SDQ) scales; parents also completed the Developmental Coordination Disorder Questionnaire (DCDQ). Results revealed a positive association of cheek cell DHA with non-verbal IQ and a negative association with teacher rating of hyperactivity and total difficulties.

#### 3.2.2. Clinical Studies (Lipid-Only Intervention)

Seven of the studies which met inclusionary criteria were randomized, clinical trials that included supplementation with either fish oil or algal oil as a source of DHA. Ryan and Nelson [37], reported results of a multi-center RCT designed to determine the effects of DHA supplementation on cognition in children. A total of 175 healthy, 4 year olds were supplemented with 400 mg DHA/day or placebo for 4 months. Cognitive function was measured using the Leiter-R test of Sustained Attention (Leiter-R SA), the Peabody Picture Vocabulary Test (PPVT), the Day-Night Stroop Test (DNST), and the Conners’ Kiddie Continuous Performance Test (Conners’ KCPT). Results showed no effect of DHA supplementation on any of the tests of cognitive function. There was a reported ceiling effect on the Leiter-R SA and the DNST in the children tested, (*i.e.*, a significant number of children had high level scores at baseline leaving little ability to show improvement on the tests) which likely influenced the results. Interestingly, a pre-planned analysis of 93 children showed a significant and positive association of whole-blood DHA and PPVT scores.

An RCT by McNamara *et al.* [12] was designed to determine the effects of DHA supplementation on functional cortical activity during sustained attention in healthy boys. A total of 33 healthy boys aged 8–10 years were supplemented with 400 or 1200 mg DHA/day, or a matching placebo for 8 weeks. The primary outcome included change from baseline in cortical activation patterns, as measured by functional magnetic resonance imaging (fMRI). fMRI scans were recorded at baseline and after 8 weeks of supplementation and whole-brain activation patterns were determined during a task of sustained attention. Results of the study confirmed that 8 weeks of supplementation with either low- or high-dose DHA significantly increased functional activation in the dorsolateral prefrontal cortex (DLPFC) during performance of the sustained-attention task (CPT-IP) as compared to placebo. The authors reported a ceiling effect for the CPT-IP task with an 80%–90% accuracy rate exhibited by all groups and, therefore, performance of the DHA-supplemented children on the CPT-IP did not differ from controls. However, the children’s erythrocyte DHA was positively correlated with DLPFC activation and inversely correlated with reaction time during the attention task.

In an RCT by Kennedy *et al.* [33], 90 healthy children aged 10–12 years were supplemented with 400 or 1000 mg DHA/day, or a matching placebo for 8 weeks. Cognition and mood were assessed using two batteries of computer-based cognitive tests, the Cognitive Drug Research Battery (CDR) and the Internet Battery. The authors reported no consistent cognitive benefit as assessed by the CDR as a result of DHA supplementation. After 8 weeks, children supplemented with 400 mg DHA/day performed faster on word recognition tasks than children given placebo while those supplemented with 1000 mg DHA/day performed slower than children given placebo. In addition, performance on the Internet Battery did not differ by treatment group except for the visual analogue measure of “relaxed”, where children supplemented with both 400 mg and 1000 mg DHA/day showed greater improvement from baseline than controls. The validity of this difference is questionable since the groups differed significantly on this measure at baseline.

The study by Dalton *et al.* [30] was a randomized, single-blind, placebo-controlled clinical study investigating the effect of a spread, rich in omega-3 fatty acids, on cognition in children. A total of 183 low-income, marginally nourished schoolchildren aged 7–9 years were given a bread spread containing marine fish flour or an analogous spread without fish flour. Children were given the spread on two slices of bread per day, on school days, for 6 months. The fish flour spread provided ~892 mg of DHA/week. The primary outcomes were change from baseline on scores from the Hopkins Verbal Learning Test battery (HVLT), a reading test, and a spelling test. After 6 months, children supplemented with fish flour spread performed better on the HVLT compared to unsupplemented children, scoring higher on measures of recognition and discrimination. Children consuming the *n*-3 spread did not experience the decline in spelling observed in the unsupplemented children over the 6 months, and there was a marginally significant benefit of supplementation on reading.

Two studies investigated the effect of omega-3 supplementation on behavior, exclusively [31,32]. Hamazaki *et al.* [31] reported results from an RCT in healthy school children from Lampung Province, Indonesia. A total of 233 children aged 9–14 years were supplemented with DHA-rich fish oil containing 650 mg DHA and 100 mg EPA/day or placebo for 3 months. Aggression and impulsivity were assessed using the Hostility-Aggression Questionnaire for Children (HAQ-C) and the Barratt Impulsiveness Scale, version 11 (BIS-11). School attendance was recorded as well. While school attendance was higher in the DHA group, no behavioral benefit as the result of supplementation was found.

Itomura *et al.* [32] reported findings from an RCT investigating the effect of omega-3 fatty acid supplementation on behavior (aggression) in healthy school children from Japan. A total of 179 healthy children aged 9–12 years were given fish oil-fortified foods (rolls, bread, sausage, and spaghetti) providing approximately 3600 mg DHA/week (~514 mg/day) plus 840 mg EPA/week (~120 mg/day) or similar unfortified foods for 3 months. Aggression was assessed using the HAQ-C and the Picture Frustration (PF) Study. Attention deficit, hyperactivity and impulsivity were assessed by parents/guardians using diagnostic questionnaires of DSM-IV for ADHD. After 3 months of supplementation, DHA-rich fish oil-fortified foods protected against increases in aggression (as assessed by the HAQ-C). Specifically, in a per-protocol analysis, HAQ-C or physical aggression, scores increased significantly between baseline and week 12 in the female placebo group, but remained unchanged in the female active treatment group. Supplementation was also associated with reduced impulsivity among females as assessed by parents/guardians. No significant changes in assessment of aggression or impulsivity were observed in males, however.

Richardson *et al.* [36] reported the results of an RCT designed to determine the effects of dietary supplementation with DHA on reading, working memory, and behavior in healthy schoolchildren. A total of 362 healthy children aged 7–9 years who were underperforming in reading (below the 33rd percentile/equivalent to ~18 months behind chronological age) were enrolled in the study. Subjects were supplemented with 600 mg DHA/day or a matching placebo for 16 weeks. The primary outcome measures were reported as change from baseline in reading, working memory, and behavior. Reading was assessed using the Word Reading Achievement sub-test of the British Ability Scale (BAS II). Working memory was assessed using two sub-tests of the BAS II (Recall of Digits Forward and Recall of Digits Backward), and behavior was assessed by parents or guardians and teachers using the Conners’ Ratings Scales (CPRS and CTRS respectively). After 16 weeks of supplementation, performance on the reading test did not differ by treatment. However, in a pre-planned analysis of 224 children with baseline reading scores ≤20th percentile, and in the subgroup of 105 children with baseline reading scores ≤10th percentile, DHA supplementation significantly improved reading. DHA supplementation also improved reading age, above the expected 4 month gain over the 16 week study period. For children with baseline readings scores ≤20th percentile, DHA supplementation led to an additional 0.8 month gain in reading age (approximately 20% greater than expected). For children with baseline reading scores ≤10th percentile, supplementation resulted in an additional 1.9 month gain in reading age (approximately 50% greater than expected. DHA also significantly improved behavior as rated by parents. Compared to children given placebo, children supplemented with DHA experienced significant improvements in eight of fourteen scales of the CPRS. No effects were seen on behavior as rated by the teachers and there were no significant effects of supplementation on memory.

#### 3.2.3. Clinical Studies (Multiple Nutrient Interventions)

Five studies included supplementation with either fish oil or algal oil as a source of DHA in addition to other fatty acids and/or micronutrients [29,34,35,38,39]. The RCT by Muthayya *et al.* [35] compared the effects of a combination of micronutrients and omega-3 fatty acids on indicators of cognitive performance in low-income, marginally nourished schoolchildren in Bangalore, India. A total of 598 children aged 6–10 years were allocated to 1 of 4 intervention groups to receive foods fortified with micronutrients at either a high (100% RDA) or low (15% RDA) level in combination with either 930 mg ALA/day plus 100 mg DHA/day (high omega-3) or 140 mg ALA/day (low omega-3) for 12 months. The four groups were defined as: high micronutrients, high omega-3; low micronutrients, high omega-3; high micronutrients, low omega-3; or, low micronutrient, low omega-3. The cognitive test battery consisted of 11 sub-tests to evaluate: fluid reasoning; short-term memory; retrieval ability; and, cognitive speediness. Outcomes were measured at baseline and after 6 and 12 months of supplementation. After 12 months, all 4 groups had significant improvements in short-term memory, retrieval ability, fluid reasoning, cognitive speediness, and overall cognitive performance. Analyses showed differences in cognitive scores according to micronutrient supplementation, but no differences were detected related to DHA or other omega-3 fatty acid supplementation.

Kirby *et al.* [34] reported the results of a one-way crossover design RCT investigating the effect of omega-3 fatty acid supplementation on cognition and behavior in healthy schoolchildren from a mainstream school population. A total of 450 healthy children aged 8–10 years were supplemented with 400 mg DHA + 56 mg EPA/day plus other micronutrients or placebo for 16 weeks. Following 16 weeks of supplementation, both groups received the active supplement for an additional 8 weeks. Outcomes included verbal and non-verbal IQ as measured by the KBIT-2, reading and spelling ability as measured by the WIAT-II, working memory as measured by the WMTB-C, attention as measured by the TEA-Ch, and visual attention and impulsivity as measured by the MFFT. In addition, handwriting process was measured using the Computerized Penmanship Evaluation Tool (ComPET), and parent- and teacher-rated behavior was assessed using the SNAP-IV and SDQ. Results showed that performance on the cognitive tests did not differ by treatment group. However, in a per-protocol analysis of the 235 children who reached compliance criteria, DHA supplementation significantly improved visual attention and impulsivity as assessed by the MFFT. The number of first correct responses was significantly higher in the active treatment group compared to the placebo group. With regard to behavior in the per-protocol analysis, supplementation protected against declines in parent-rated pro-social behavior. In the intention-to-treat analysis, however, teachers saw improvement in the “difficulties” score in unsupplemented children only.

Sinn *et al.* [38] reported results from an open-label pilot study designed to investigate the feasibility of providing fish oil supplements to children in a remote, Northern Territory school in Australia. A total of 47 children, aged 3–14 years, were given a supplement during school days providing 558 mg EPA + 174 mg DHA, plus 60 mg GLA, plus 10.8 mg vitamin E/day for 12 weeks. Reading and spelling were assessed using the Wide Range Achievement Test (WRAT); non-verbal intelligence was assessed using the Raven’s Colored Matrices; and behavior was assessed by teachers using the Conners’ Behavior Ratings Scales (CBRS). Outcomes were measured at baseline and after 12 weeks. After 12 weeks of supplementation, reading and spelling scores and performance on the Raven’s Coloured Matrices improved significantly. It is important to note, however, that this pilot study was not placebo-controlled and the observed changes may not be due to supplementation.

The Nutrition Enhancement for Mental Optimization (NEMO) study group [39] reported results of a RCT which assessed the effect of micronutrients, long-chain omega-3 fatty acids, or both on cognitive performance in well-nourished and marginally nourished children age 6–10 years. A total of 396 well-nourished children from Adelaide, South Australia, and a total of 384 marginally nourished children from Jakarta, Indonesia, were enrolled in the study. Children received a fruit-flavored drink containing: a micronutrient mix alone; an omega-3 fatty acid mix alone; a combination of the micronutrient and omega-3 fatty acid mixes; or a placebo mix. The fruit-flavored drink was consumed 6 days/week for 12 months. The micronutrient mix provided 100% of the RDA for iron, folate, vitamin B6, vitamin B12, vitamin A, and vitamin C and 50% of the RDA for zinc. The omega-3 fatty acid mix provided 88 mg DHA/day and 22 mg EPA/day. Cognitive function and school performance were assessed after 6 and 12 months of supplementation using a series of standardized neuropsychological tests. Subjects from the two test sites were different with regard to several characteristics including nutritional status; therefore data for Indonesia and Australia were analyzed separately. From the Australian site, results showed that in well-nourished, school-aged children, 12 months of supplementation with multiple micronutrients improved verbal learning and memory, but had no effect on tests of general intelligence or attention. Supplementation with omega-3 fatty acids did not affect cognitive function or school performance. In the Indonesian children who were marginally nourished, results showed that fortification with multiple micronutrients improved verbal learning and memory in girls but not in boys. As in the Australian cohort, omega-3 fatty acids had no effect on any measure of cognitive function or school performance.

Baumgartner *et al.* [29] reported the findings of an RCT investigating the effects of iron and DHA/EPA supplementation alone and in combination, in children with poor iron and omega-3 fatty acid status. A total of 321 children, aged 6–11 years, with iron deficiency were allocated to receive iron, DHA/EPA, iron plus DHA/EPA, or a matching placebo 4 days/week for 34 weeks. Iron was provided at a dose of 50 mg/day and fish oil was provided at a dose of 420 mg DHA/day and 80 mg EPA/day. Because supplementation was interrupted by holidays and teacher strikes, supplements were provided for 105 days. Mean total iron intake over the 105 day supplementation period was 4.8 g (45.7 mg/day). Mean total DHA intake was 41.2 g (392 mg/day), and mean total EPA intake was 7.8 g (74 mg/day). The primary outcome measures were change from baseline in tests of cognition including 4 subtests of the Kaufman Assessment Battery for Children (KABC-II) and the Hopkins Verbal Learning Test (HVLT). Results of the study indicated that while children supplemented with iron performed better than un-supplemented children on the HVLT test of learning and memory, DHA/EPA supplementation had no effect on learning or memory. Results of the study also found that in a sub-group analysis stratified by anemia status, children with iron deficiency anemia supplemented with DHA/EPA performed significantly worse than children given placebo on the KABC-II test of long-term memory. In a second sub-group analysis stratified by sex, there was a significant negative effect of DHA/EPA supplementation on the Atlantis Delayed test performance (long term memory and retrieval) in girls, while boys benefitted from supplementation on this measure. On the HVLT, girls receiving DHA/EPA supplementation alone had better recall than placebo or those receiving the combination with iron.

## 4. Discussion

Infancy, childhood, and adolescence are times of rapid neuronal maturation, synaptogenesis, and gray matter expansion, all of which are associated with brain DHA accumulation [5,13]. DHA synthesis from its fatty acid precursors is known to be inefficient, making dietary sources of preformed DHA important [41]. However, DHA is found at high levels in only a very limited number of foods and the typical intake by children worldwide is surprisingly low.

Studies in animals show that omega-3 deficiency causes structural and functional changes in the hippocampus, hypothalamus, and cortex areas of the brain [18,42,43]. In animals, reduced levels of brain DHA are associated with problems of spatial and serial learning and memory [42,44] as well as increases in depressive symptoms and aggressive behavior [45,46]. In humans, DHA is particularly important during gestation and infancy for early brain and visual development [47,48]. There is also growing understanding of the role that *n*-3 LC-PUFA may play in the prevention and treatment of several neuropsychiatric conditions common to children and adults [49,50]. Recently, polyunsaturated fatty acids, including DHA, have been studied in children with certain developmental disorders characterized by learning and behavior difficulties. Many of these conditions are associated with abnormal fatty acid status, and whether due to altered fatty acid metabolism or poor intake, some studies show an improvement in symptoms following supplementation with polyunsaturated fatty acids [22,51,52]. Healthy children without these neuropsychiatric conditions may also have poor DHA status, due to poor intake. It is therefore plausible that improvements in DHA status could benefit learning and behavior in healthy children as well. The purpose of this review was to determine whether current literature defines a relationship of DHA with learning and behavior in healthy children.

Although there appears to be interest in investigating the effects of DHA and other fatty acids on cognition, learning and behavior in healthy children, the results of our search revealed studies which were very diverse in both research design and focus. For assessment of cognition, the investigators of studies in this review used many and varied age-standardized tests, most of which contained multiple-subtests each measuring a different aspect of cognitive function. There were no apparent consistent results among the studies with regard to treatment effects or DHA biomarker status on specific cognitive tests. In spite of this heterogeneity, however, the studies as a group provide a global view of DHA impact on brain activity and cognition, with over half of the studies reporting significant results favoring a role of DHA or *n*-3 LC-PUFA in at least one area of cognition, learning, or behavior.

Two studies included in this review, demonstrated a benefit of DHA by direct measurement of brain activity using neurophysiological measures of cognitive function during performance of a standardized test. First, the study by McNamara *et al.* [12] showed the effects of DHA supplementation on brain activity in children as measured by fMRI during performance of a task of sustained attention. Even though outcomes on the standardized task showed no difference as a result of supplementation, probably due to a ceiling effect for the CPT-IP in the children, fMRI data confirmed enhanced activation of the dorsolateral prefrontal cortex (DLPFC) in the DHA-supplemented children. There is a normal age-related increase in DHA content in the prefrontal cortex which occurs during adolescence and young adulthood in healthy well-nourished subjects [13] and the study by McNamara *et al*. [12] provides a better understanding of the extent to which apparently well-nourished youth may be deficient in DHA and how this deficiency can affect brain activity. Second, a study by Boucher *et al.* used electroencephalographic data to directly assess brain activity in healthy children. In that study, ERP results showed a significant positive association of DHA status with brain activity during performance of a standardized test [27]. While the association with ERP outcomes was stronger for cord blood DHA status than for the child’s current plasma DHA, it was still apparent that DHA status in the 10–13 year old children was not optimal and that brain activity was, to some degree, correctable or modifiable through supplementation. There was no effect of DHA status on the outcome of the standardized test.

The findings of McNamara *et al.* [12] and Boucher *et al.* [27] are supported by a similar study in an older population. Jackson *et al.* used near-infrared spectroscopy to assess the relative changes in concentration of oxyhemoglobin and deoxyhemoglobin in the prefrontal cortex of young adults during performance of standardized cognitive tasks [53]. In this study, supplementation with 1- or 2-g DHA-rich fish oil increased concentrations of oxyhemoglobin and total hemoglobin in participants during completion of a standardized test. This pattern of oxygenation is indicative of increased blood flow. As in the studies by Boucher *et al.* [27] and McNamara *et al.* [12], there were no consistent changes in performance on the cognitive test in spite of the changes in the hemodynamic response.

Studies such as those by Boucher *et al*. [27], McNamara *et al.* [12], and Jackson *et al.* [52] which demonstrate a DHA relationship with measurable brain activity, but without a concurrent measurable effect on test performance, bring into question the appropriateness or sensitivity of many commonly administered cognitive tests used for assessing DHA adequacy. It is possible that certain tests do not assess the particular cognitive activity affected by DHA. In addition, it is more likely that larger group sizes are required in order achieve the statistical power needed for valid comparisons. From our review, it is interesting that DHA-related improvements in school performance were, however, detectable in some of the studies. In particular, reading performance improved in DHA-supplemented children with poor reading skills in the study by Richardson *et al.* [36]; and in the study by Dalton *et al.* [30], spelling performance was maintained in a DHA-supplemented group while the control group experienced a loss of skills. Functions of the brain are often described in modular terms and the type of learning detected in these two studies may involve a coordination of several functions, including memory and attention. Providing adequate DHA may provide small improvements in multiple areas of cognitive function which are ultimately sufficient to affect reading or spelling.

The implication of improving school performance in reading or spelling for struggling students is significant. Reading is described as the major foundational skill for all school-based learning. Students who do not achieve proficiency in reading during the early years of school are known to have difficulty comprehending subject matter in the grades that follow. Educational experts suggest that when students have difficulty learning to read, their love of learning and motivation diminishes [54]. In addition, experts define reading as a critical skill needed not only for academic success, but also for social and economic success [55]. Children with poor reading skills have been shown to experience decreases in self-esteem, self-concept, and further motivation for learning [56] and are more likely to become frustrated, overwhelmed, or disinterested [57]. Given the importance of academic success to children’s well-being and ultimate success in life, all educational and environmental factors, including improved nutrition, are worthy of consideration for optimization of learning.

Since changes in normal, healthy children may be difficult to detect, the study design, subject selection, and outcome measure selection are increasingly important. In the study by Richardson *et al.* [36], the authors state that the original trial design required children to rank below the 20th percentile (2 years behind actual age) in reading. However, recruitment concerns led the investigators to broaden the inclusion criteria to those scoring below the 33rd percentile (18 months behind actual age) in reading with a pre-planned analysis of children below the 20th and 10th percentile on reading. While no treatment effect was detectable in the whole population, a significant effect on the change from baseline reading scores was detected in children scoring below the 20th percentile and an apparent greater and significant effect was observed for those who scored below the 10th percentile at baseline. Similar findings were reported in a smaller study conducted in children with ADHD which included a subgroup of children who were also behind in their age level in reading and spelling by approximately 2 years. Following 4 months of supplementation with either high EPA or high DHA fish oil, no group differences in behavior or cognition were detected in the group as a whole. However, higher erythrocyte DHA levels were associated with improved word reading, spelling, attention, and oppositional behavior, particularly in the subgroup with reading and spelling difficulties [58].

The doses of DHA supplements used in the studies included in this review varied widely. The 600 mg daily dose of DHA used by Richardson *et al.* [36] is similar to that used in studies of children with learning disorders; it establishes an effective dose for use in healthy children as well. Whether 600 mg DHA/day is the optimal dose for healthy children of this age remains to be tested. Future studies are needed to define blood DHA levels, as a surrogate for brain DHA level, which are associated with both the low and improved scores on tests of reading and spelling. Due to differences in study design, methods of measuring and reporting blood DHA, level of DHA supplementation, and compliance, the current literature is still incomplete in this regard. In the study by McNamara *et al.* [12], mean erythrocyte DHA composition at baseline was 3.3% of total fatty acids. Following supplementation with 400 mg DHA/day or 1200 mg DHA/day for 8 weeks, erythrocyte DHA composition increased to 7.5% and 10.3%, respectively. Since these levels were associated with activation of the dorsolateral prefrontal cortex, they could be considered as appropriate goals for future RCTs. Only four other RCTs reported erythrocyte DHA composition as seen in Table 3 [29,31,32,35]. Of these, the studies by Itomura *et al*. [32] and Hamazaki *et al.* [31] achieved erythrocyte DHA levels of 7.1% and 7.5% total fatty acids, respectively. Baumgartner *et al*. [29] and Muthayya *et al*. [35] reported erythrocyte DHA levels of less than 6% following the supplementation period. The remaining studies either did not report DHA blood status or reported DHA status in a different blood compartment or tissue, making comparisons questionable.

The oils used as placebo in the included studies varied greatly with regard to fatty acid composition. Some, such as medium chain triglycerides provided no additional *n*-6 or *n-*3 PUFA while others provided additional linoleic acid from corn or sunflower oil, ALA from soybean and rapeseed oil, or monounsaturated fatty acids and polyphenols from olive oil. The search for the most appropriate placebo for both human and animal studies has been a topic of debate, with virtually all substitute oils providing fatty acids or other nutrients which on their own can be considered bioactive [59]. For studies specifically designed to increase blood and tissue levels of DHA, oils which supply additional *n*-3 fatty acids in the form of ALA may have minimal effect due to poor conversion of ALA to DHA in humans [41]. However, oils that contribute significant linoleic acid to the diet may reduce DHA accrual in tissues. Studies in animals show decreased incorporation of DHA in retinal tissue in diets high in linoleic acid, and that diets low in linoleic acid support higher brain DHA [60,61]. None of the studies in this review reported fatty acid composition of the background diet; therefore it is not clear whether the small amount of oil used in supplementation significantly impacted fatty acid intake of ALA or LA.

In addition to the dose and duration of DHA supplementation, many studies suggest that blood and tissue biomarkers of *n*-3 PUFAs are associated with several common single nucleotide polymorphisms (SNPs) in the genes responsible for encoding the delta 5, and delta 6 desaturases (FADS1 and FADS2), as well as ELOVL2 elongase. Because there is a distinct association of the haplotypes with both *n*-3 and *n*-6 PUFA biomarker status, individuals with variations of these mutations respond differently to *n*-3 supplementation as compared to the larger population. The fatty acid profile associated with SNPs in the FADS1-FADS2 gene cluster includes increased proportions of precursors such as ALA and decreased proportions of desaturation products such as EPA and DHA. However, the biosynthesis of DHA is very limited in the general population and direct consumption of DHA is the primary way to increase DHA status making distinction between the various haplotypes of less significance for this fatty acid. Large observational studies as well as metabolic tracer studies of ALA metabolism in adults indicate that polymorphisms in the FADS1-FADS2 gene cluster do not result in changes in plasma DHA composition under conditions of a controlled diet. Since this review focused on studies which specifically modified dietary DHA, and not its precursors, such genetic variations would be expected to play only a minor role on their outcomes [62,63,64,65,66].

The criteria for inclusion in this review specified that studies report a biomarker of the children’s DHA status from the same time-period as the cognitive or behavioral testing. Studies in animals, however, suggest that earlier periods of development may represent a more critical window of time when adequate DHA is particularly essential. Such findings indicate that *n*-3 deficient diets limit DHA accrual during the perinatal period, and if not corrected early, are associated with deficits in neuronal arborization and synaptic formation. Deficient accrual of brain DHA is also associated with deficits in dopamine and serotonin neurotransmission. Development of these two systems of neurotransmission is of particular interest due to their well-known involvement in the control of learning and memory. In a series of studies using a rodent model, Chalon *et al.* [67] demonstrated that brain DHA decreased in those fed an *n*-3 deficient diet and that this was accompanied by a corresponding decrease in the amount of dopamine in the frontal cortex. Animals fed an *n*-3 repletion diet at birth recovered to normal levels of both brain DHA and dopamine. However, a delay in feeding the *n*-3 repletion diet to the period following weaning resulted in only partial recovery of brain DHA and no recovery of the neurochemical factors. The implication is that early and prolonged insults from a severe *n*-3 deficiency may lead to irreversible damage to specific brain functions. While no critical window of development has been defined in humans, it is possible that differences in early DHA status affect the outcomes of these studies in later childhood [67,68].

## 5. Conclusions

In summary, the studies included in this review generally indicate that improvements in DHA status may initiate brain changes which are observable in activities of learning and behavior. Results of neurophysiologic measures that directly assessed brain activity indicate that brain changes occur in healthy children as the result of DHA supplementation. However, standardized tests of cognition do not show consistent changes. The improvement seen in reading and spelling skills following DHA supplementation may represent an accumulation of many subtle changes made over multiple domains that are not readily detectable on other types of tests. These changes may be particularly subtle in healthy children. The reviewed studies conducted in healthy children build on animal data that demonstrate DHA is an essential brain component affecting learning and behavior. While the number of studies is limited, and the design features are diverse, the studies implicate problems in learning and behavior as detrimental effects of DHA deficiency in otherwise healthy children.
